# A Composite Network Approach for Assessing Multi-Species Connectivity: An Application to Road Defragmentation Prioritisation

**DOI:** 10.1371/journal.pone.0164794

**Published:** 2016-10-21

**Authors:** Luca Santini, Santiago Saura, Carlo Rondinini

**Affiliations:** 1 Department of Environmental Science, Institute for Wetland and Water Research, Faculty of Science, Radboud University, P.O. Box 9010, NL-6500 GL, Nijmegen, The Netherlands; 2 Global Mammal Assessment Program, Department of Biology and Biotechnologies, Sapienza Università di Roma, Viale dell’Università 32, 00185, Rome, Italy; 3 European Commission, Joint Research Centre (JRC), Directorate for Sustainable Resources, Land Resources Unit, Via Enrico Fermi 2749, I-21027, Ispra (VA), Italy; Centre for Cellular and Molecular Biology, INDIA

## Abstract

One of the biggest challenges in large-scale conservation is quantifying connectivity at broad geographic scales and for a large set of species. Because connectivity analyses can be computationally intensive, and the planning process quite complex when multiple taxa are involved, assessing connectivity at large spatial extents for many species turns to be often intractable. Such limitation results in that conducted assessments are often partial by focusing on a few key species only, or are generic by considering a range of dispersal distances and a fixed set of areas to connect that are not directly linked to the actual spatial distribution or mobility of particular species. By using a graph theory framework, here we propose an approach to reduce computational effort and effectively consider large assemblages of species in obtaining multi-species connectivity priorities. We demonstrate the potential of the approach by identifying defragmentation priorities in the Italian road network focusing on medium and large terrestrial mammals. We show that by combining probabilistic species graphs prior to conducting the network analysis (i) it is possible to analyse connectivity once for all species simultaneously, obtaining conservation or restoration priorities that apply for the entire species assemblage; and that (ii) those priorities are well aligned with the ones that would be obtained by aggregating the results of separate connectivity analysis for each of the individual species. This approach offers great opportunities to extend connectivity assessments to large assemblages of species and broad geographic scales.

## Introduction

Natural areas have largely been converted to croplands, plantations, pastures and human-made infrastructures [1], increasing the fragmentation of natural habitats globally [2] and negatively affecting species richness and abundance [3]. Europe is one of the continents with the longest history of land use [4], and today a large proportion of European lands are devoted to agriculture [5]. Europe is also densely populated and highly urbanized [5,6], and holds one of the densest road networks globally [7]. Roads are indeed one of the most disrupting elements in landscapes, likely limiting animal movements and spatial dynamics of natural populations. Specifically, roads are known to have important ecological effects on wildlife through reduced habitat quality, increased mortality due to collisions with vehicles, and disruption of movement patterns potentially leading to genetic isolation [8–11]. European habitats are therefore considered to be highly fragmented [5,6].

A significant achievement for European habitat conservation and connectivity has been the implementation and expansion of the Natura 2000 network of protected areas, today covering about 18% of the land area of the European Union (EU) with its current 28 member states [12]. Additionally, under the EU Biodiversity Strategy to 2020, EU committed to six targets, among which target II aims to implement Green Infrastructures and restore at least 15% of Europe’s degraded ecosystems by 2020 [13]. Green Infrastructures include natural and man-made structures and solutions that aim to facilitate the flow of ecosystem services and conserve biodiversity [14]. Examples of green infrastructure span from natural areas, ecological corridors, wildlife friendly farming, green roofs and walls, and wildlife crossings (underpasses and overpasses). Wildlife passes are interventions aimed to reduce the barrier effect of roads, in order to increase connectivity for terrestrial animals and reduce mortality due to collisions with vehicles. A vast literature reports wildlife use of crossings structures, but only in few cases wildlife monitoring has provided clear evidence of their benefits, as this would require pre- and post-construction monitoring studies recording wildlife dispersal rates and genetic isolation [15,16]. Wildlife crossings are usually expensive, and—as all conservation investments—need a prioritisation scheme and a clear framework to be applied in order to identify potential spots where connectivity is expected to benefit following their implementation. By controlling for the effect of confounding variables, Clevenger and Waltho [17] demonstrated the importance of structural aspects in affecting species use of crossing structures, as well as species-specific differential use.

A number of studies have developed or applied methods to identify areas in which to implement connectivity restoration measures, either focusing on a particular species [18,19], or on a more generic landscape resistance surface through which the potential movement pathways are identified [20–24]. These studies do not explicitly consider multiple species existing in a community, and hence the restoration priorities they propose are valid only for one particular species, or, when focusing on more generic assessments on the effect of the resistance matrix, they disregard the actual species distribution and species-specific habitat use, and hence provide partial or incomplete answers for green infrastructure design. Focusing on species assemblages, rather than on just one or a few individual species as in common conservation plans, while still accounting for the differential distribution of each species and keeping the analysis tractable from a computational and practical perspective, is required for green infrastructure planning. Such as an assemblage-level planning needs to consider as broadly as possible the biodiversity that may be affected by grey infrastructure and benefited by connectivity restoration and road defragmentation measures.

The identification of areas where to implement green infrastructures should be based on the value of these areas for wildlife crossing [18], and thus should be a function of wildlife habitat use, estimated current connectivity, and the estimated potential connectivity increase after restoration intervention. Ideally, such infrastructure should benefit the largest number of species possible. For regional or national planning, it would be out of scope and computationally impossible to identify exact spots where crossing infrastructure should be implemented, given that (i) there might be many thousands of alternative locations to be examined and (ii) subsequent finer-scale studies would need to be conducted to provide more precise locations within the coarser-scales priority areas identified at the national level. Therefore, for national, continental or regional planning, analyses need to be conducted at a relatively coarse resolution, not examining each individual road sector but areas with potentially different impacts of roads on connectivity.

One of the most widely applied approaches in connectivity conservation is graph theory, which models a set of habitat patches as a network of nodes and links (also known as edges) with various degrees of connectivity [25–27]. Generally, nodes are characterized by an attribute (weight) that represents some ecological aspect of patches such as habitat quantity or quality, which is assumed to be related with local population size (actual or potential), whereas links may be represented by the probability of crossing the matrix between two nodes given species' dispersal abilities, distance between nodes and matrix permeability. Most of previous applications of graph theory for connectivity have focused on single or few species or on a range of dispersal distances [12,18–21,28–30]. It remains however untested if the use of graph theory for connectivity analysis, given its strong demands on computational resources for large networks, is a practical and tractable approach in the challenging situation in which not just large areas but many different species are to be simultaneously considered and integrated in the delivered conservation guidelines.

Here we show how species-specific graphs can be aggregated in order to account for many species in a single composite graph, and still delivering results that are very similar to those that would be obtained by conducting connectivity analyses separately for each species. In this way, the proposed network aggregation approach is able to considerably reduce computational times and the analytical complexity of the planning process without affecting the final results. Specifically, we focus on an application example to illustrate the potential of this approach, by identifying areas eligible for implementing wildlife crossings in the Italian territory considering 20 Italian terrestrial mammal species. We build an ecological network for each individual species, and generate eight alternative composite networks to test the reliability of different network aggregation approaches. On this basis, we identify the areas that, if restored, would maximise the potential increase in connectivity for the species assemblage as a whole, and thus identify priorities for restoration and implementation of wildlife crossings. The proposed approach can be applied to different connectivity studies or planning situations involving a large number of species for which distribution, habitat use and approximate dispersal distances are known or can be estimated, and not only to identify the specific type of connectivity restoration priorities here considered.

## Methods

### Italian ecological network

We used biological trait data from PanTHERIA database [31] to estimate the median dispersal distance for all 64 Italian non-volant terrestrial mammal species. We used the statistical models in Whitmee and Orme [32] when body mass, home range area, population density and weaning age were known; and the models in Santini *et al*. [33] when only home range or body mass and diet category were available (S1 Table). Given that we focused on nation-wide analyses with a spatial resolution of 10 km cells (see below), we restricted the analyses to those species for which dispersal had some considerable likelihood of occurring at a distance of at least 10 km (the distance separating the closest adjacent cells). Therefore, we only considered species with a median dispersal distance of at least 3 km, which according to a negative exponential kernel corresponds to a probability of 0.1 of covering 10 km. This restricted our sample to 20 species (S1 Table). The same approach described below could however be applied to more local scales at higher spatial resolutions in which species with lower mobility can be considered.

We represented the ecological network of terrestrial mammals in Italy as a raster with a spatial resolution of 10 km. We created a graph where each 10-km cell acted as a node directly linked to the eight adjacent cells (2,982 nodes in total). The resolution of 10 km was chosen as it was considered appropriate to indicate nation-wide restoration priorities and to allow for computational efficiency of the demanding analyses of multiple networks to be performed, as these were conducted both at the aggregated and individual species levels. Previous research indicated that connectivity priorities identified by the same connectivity metrics used in this study are robust to the variation in analytical resolution [34]. We used habitat suitability models from Rondinini *et al*. [35] as distribution proxies of the 20 considered terrestrial mammals occurring in Italy. These habitat suitability models are restricted to each species' geographic range, have a resolution of 300 m, and assign habitat types one of three levels of suitability: high suitability that represents the primary habitat of the species, medium suitability where the species can be found only if nearby highly suitable habitat exists, and low suitability where the species can only be occasionally found.

We converted the habitat suitability maps to 1 (high suitability) or 0 (medium or low suitability), in order to focus on the connectivity among the highest-quality areas more likely to support species presence and reproduction in the long term. Medium-suitability habitat by definition can be frequently visited but does not host reproductive populations [35]. The 300-m resolution suitability values were summed within each 10-km node to obtain a map of habitat suitability for each of the terrestrial mammals in Italy at the spatial resolution of 10 km. We used the sum of species suitable habitat to weight each node in the graph. To weight the graph links, we estimated the probability of dispersal among landscape's adjacent cells as a function of the median dispersal distance of species (mDisp) and the distance between the centroids of the two cells (CD), using a negative exponential dispersal kernel as follows:

$$\text{Probability of dispersal}=\text{exp}\left(\frac{\text{ln}\left(0.5\right)}{\text{mDisp}}*\text{ CD}\right)$$ (1)

Where $–\frac{mDisp}{ln\left(0.5\right)\;}$ equals the mean dispersal distance in the negative exponential kernel. CD was equal to 10 km when the cells were orthogonally adjacent, and to 10*√2 km when diagonally adjacent.

### Accounting for roads

We downloaded the road layer for Italy from http://download.geofabrik.de/europe.html (accessed on May 2015), and filtered it to include only highways, primary, and secondary roads, plus all respective road links, and excluded all roads reported to be in tunnels or on bridges. As road density has been suggested as a good proxy of wildlife impact [8], the layer was first rasterized at 300 m resolution and then resampled at 10 km resolution by calculating mean values, thus obtaining a raster representing the density of roads by cell. For the purpose of the study, we assumed that the probability of dispersal between two cells decreased as a negative exponential function of the mean road density of the two cells, where the median road density of all cells in the landscape corresponded to a 50% reduction of the probability of dispersal between two cells. We assumed a negative exponential decrease as the distribution of road density was highly skewed on the left (many low road density values and a few very high). Therefore the final probability of dispersal between two cells *i* and *j* (*p*_*ij*_) was the product of the dispersal probability (Eq 1) and of the road density in the landscape matrix separating the centre of the cells.

### Ecological network connectivity and restoration priorities

For each species we measured landscape connectivity using the Probability of Connectivity metric (PC) [27,36], a graph-based habitat availability (reachability) metric that expresses the probability that two random points in the landscape fall into habitat areas that are interconnected.

$$\text{PC}=\frac{\sum_{\text{i}=1}^{\text{n}}\sum_{\text{j}=1}^{\text{n}}{\text{a}}_{\text{i}}{\text{a}}_{\text{j}}{\text{p}}_{\text{ij}}^{*}}{{\text{A}}_{\text{L}}^{2}}$$ (2)

Where *n* is the total number of nodes in the graph (here 10 km cells), *ai* and *aj* are the attributes of cells *i* and *j* (amount of suitable habitat), p*ij is the maximum product probability of dispersal among them (considering the most likely pathway using both direct and indirect connections), and *A*L is the maximum landscape attribute (here total landscape area, i.e. area of Italy).

To assess the contribution of potential interventions to restore connectivity by using green infrastructures such as wildlife passes (restoration priorities), we calculated the absolute increase of overall landscape connectivity (PC value) following removal of the barrier effect of roads in each cell in the network, indicating in which areas the improvement of connectivity would be more beneficial. This increase was quantified as *varPC*, similarly as done by Saura & Rubio [36] for quantifying the absolute decrease in connectivity by the removal of a node from the network, but here focusing instead on the connectivity increase from the removal of the barrier effect of roads in a given cell:

$$varPC_{k}=PC_{restored,k}-PC_{ini}$$ (3)

where *PC*_*restored*,*k*_ is the value of PC that results after replacing the probability of dispersal from an individual cell *k* to all the eight adjacent cells by the probability of dispersal that would result if all roads in the 9 cells were made fully permeable (full mitigation of their barrier effect), which is equivalent to the hypothetical optimum case (in terms of species connectivity) of zero road density in those cells. *PC*_*ini*_ is the value of PC in the initial (current) landscape network (with all roads as currently existing). The value of *varPC*_*k*_ hence represents the absolute increase of PC after the removal of the barrier effect of all roads from the focal node (cell) *k* to the 8 adjacent cells.

The restoration priorities (*varPC*_*k*_) were calculated for each cell in each of the individual species networks and in the different multi-species composite networks described next.

### Composite networks and aggregation of species results

We considered 2 alternative aggregations of the node attributes for each individual species *k* (*a*_*i*,*k*_) into the node attributes of the composite multi-species network ($\bar{a_{i}}$), as given by Eq 4 (sum of node attributes for all species) and 5 (square root of the sum of squared attributes for all species):

$$\bar{a_{i}}=\sum_{k=1}^{n}a_{i,k}$$ (4)

$$\bar{a_{i}}=\sqrt{\sum_{k=1}^{n}a_{i,k}^{2}}$$ (5)

Where *n* is the total number of species (here *n* = 20 for the case study on the Italian ecological network). Eq 5 was used because this would give exactly the same value of the PC index (see Eq 2 above) for the composite network and the cumulative results (sum of the PC values calculated separately for each individual species) in the particular extreme case in which all cells were completely isolated from each other, i.e. zero dispersal distance, only intra-patch (intra-cell) connected habitat would exist (this is the case in which the PC values are only influenced by the node attributes).

We explored 4 alternative aggregations of the individual species link probabilities for each species *k* (*p*_*ij*,*k*_) into link probabilities for the composite multi-species network ($\bar{p_{ij}}$). The first aggregation consisted only in an average of all link probabilities across species (Eq 6), while the other three aggregations also considered the suitable habitat area for species *k* in a given cell *i* (a_*i*,*k*_) but in different ways when calculating the aggregated probabilities (Eqs 7–9). Eq 7 weighted the average of the probabilities by the average suitable habitat area in the two nodes *i* and *j*. Eq 8 weighted the average of the probabilities by the product of the suitable habitat areas in the two nodes *i* and *j*, which is in fact the same type of weighting used in the PC metric (see Eq 2 above). Eq 9 differed from Eq 8 in that in the denominator it used the node attribute aggregation described above in Eq 5, i.e. that corresponding to intra-patch connectivity only (no dispersal between different cells).

$$\bar{p_{ij}}=\frac{\sum_{k=1}^{n}p_{ij,k}}{n}$$ (6)

$$\bar{p_{ij}}=\frac{\sum_{k=1}^{n}\left(\left(a_{i,k}+a_{j,k}\right)/2\right)\cdot p_{ij,k}}{\sum_{k=1}^{n}\left(a_{i,k}+a_{j,k}\right)/2}$$ (7)

$$\bar{p_{ij}}=\frac{\sum_{k=1}^{n}a_{i,k}\cdot a_{j,k}\cdot p_{ij,k}}{\sum_{k=1}^{n}a_{i,k}\cdot a_{j,k}}$$ (8)

$$\bar{p_{ij}}=\frac{\sum_{k=1}^{n}a_{i,k}\cdot a_{j,k}\cdot p_{ij,k}}{\sqrt{\sum_{k=1}^{n}a_{i}^{2}}\cdot \sqrt{\sum_{k=1}^{n}a_{j}^{2}}}$$ (9)

In all cases, missing links between two nodes for individual species (when a species is absent from at least one of the two nodes) were assumed to have a *p*_*ij*_ equal to zero. The combination of 4 types of aggregation for the links and 2 for the nodes resulted in 8 different composite networks for Italy. A summary of aggregation types tested is presented in Table 1.

**Table 1:** Aggregation types of individual species networks into a single composite network, and their correlation coefficient with the sum of individual species’ restoration priorities (*varPC*). See methods for further details on the aggregation procedures for nodes and links.

Aggregation type	Node attributes	Link probabilities	Spearman's r
**A**	Node attribute sum (Eq 4)	Mean (Eq 6)	0.906
**B**	Node attribute sum (Eq 4)	Mean weighted by mean habitat (Eq 7)	0.968
**C**	Node attribute sum (Eq 4)	Mean weighted by habitat product (Eq 8)	0.966
**D**	Node attribute sum (Eq 4)	Mean weighted by habitat product but normalized by intra-patch connectivity (Eq 9)	0.966
**E**	Sum of the intra-patch connectivity (Eq 5)	Mean (Eq 6)	0.925
**F**	Sum of the intra-patch connectivity (Eq 5)	Mean weighted by mean habitat (Eq 7)	0.976
**G**	Sum of the intra-patch connectivity (Eq 5)	Mean weighted by habitat product (Eq 8)	0.965
**H**	Sum of the intra-patch connectivity (Eq 5)	Mean weighted by habitat product but normalized by intra-patch connectivity (Eq 9)	0.966

We compared the restoration importance of each cell (*varPC*) in the 8 composite networks with the importance calculated by summing in each cell the *varPC* results for each of the 20 species (sum of *varPC* of all species; hereafter referred to as “Cumulative results”). We selected the composite network showing the highest Spearman's correlation coefficient with the cumulative results.

Then we explored which species contributed the most to the results (*varPC* values) of the aggregated networks (i.e. composite and cumulative). We divided the *varPC* of each species per cell by that of each aggregated network, and then averaged it per species in order get a mean proportion of *varPC* per species per aggregated network. To disentangle which characteristics made species more influencing, we regressed the mean proportion of *varPC* per species, with species median dispersal distance and amount of habitat (expressed as mean proportion of suitable habitat per cell). These two predictive variables were standardized to a mean of zero and SD of one to compare their slopes (relative contribution to the mean proportion of *varPC*).

Because different restoration costs would be required for different cells, we also calculated restoration priorities standardized by road density (*varPC* values divided by road density) by assuming that restoration effort is proportional to road density in the cell. This would also approximately correspond to the priority obtained by simulating smaller, but even, restoration actions across cells.

All connectivity analyses were run on a new command line version for Linux of Conefor Sensinode 2.6 ([37]; updated at www.conefor.org). GIS and additional analyses were performed in GRASS 7.0 [38] and R 3.0.3 [39] using the package 'Raster' [40].

## Results

**Fig 1:**
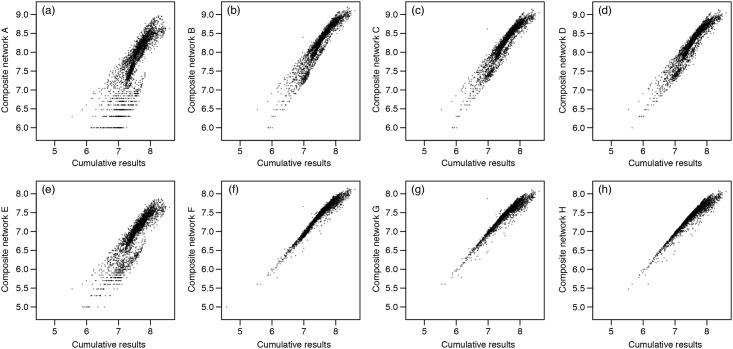
Relationship between restoration priority values (*varPC*) obtained from the cumulative results (sum of individual species restoration priorities) and the 8 composite networks. See Table 1 for the description of the different aggregation approaches used in producing these composite networks.

**Fig 2:**
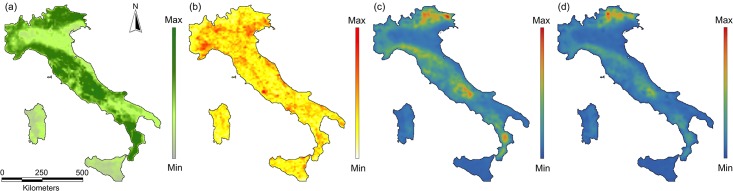
(a) Amount of suitable habitat (node weight), (b) Road density (used for obtaining the link weights), (c) restoration priority as given by *varPC* values (cells where actions to mitigate the barrier effect of roads would yield the highest benefit) according to the cumulative results (sum of individual species restoration priorities), and (d) restoration priority according to the best performing composite network (composite network F).

**Fig 3:**
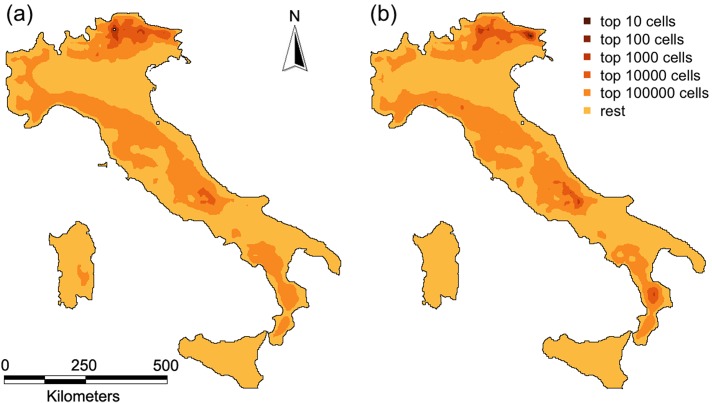
Restoration priorities for (a) the cumulative results and (b) the best composite network, both classified in different area targets (10; 100; 1,000; 10,000; 100,000 cells).

The cell restoration priorities obtained using the composite network provided results that were in general highly correlated with the priorities for the cumulative results from individual species, although the alternative methods for aggregating the networks performed differently (Fig 1; Table 1). The best performing composite network (Composite network F; Table 1) was obtained when node attributes were given by an approximation of the intra-patch connectivity (Eq 2), and when the probabilities in the composite links were calculated as the average of the probability of dispersal weighted by the amount of habitat in the two cells for each species (Eq 7), which resulted in a Spearman's r = 0.976 (Fig 1; Table 1). The direct display of *varPC* values (restoration priorities) showed some slight but noticeable differences between the best composite network and the cumulative results from individual species networks (Fig 2) because of the different statistical distributions of the *varPC* values. These differences, however, decreased even further when the rank of cells by *varPC*, rather than the actual *varPC* values, was considered (Fig 3); given increasing area targets for restoration, the location of the areas identified for the cumulative results and the best composite network were very similar (Fig 3).

**Fig 4:**
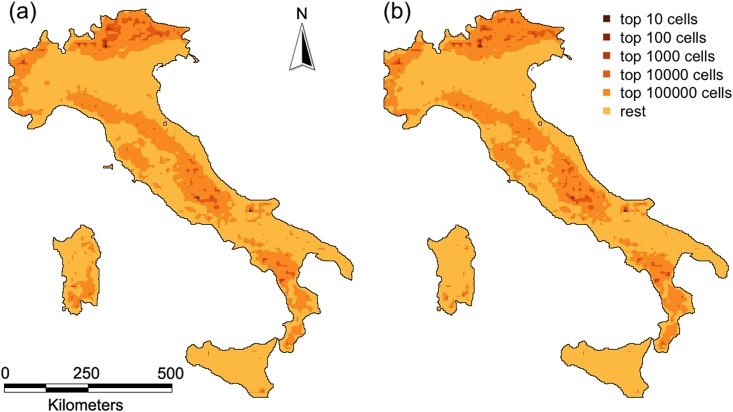
Restoration priorities normalized by road density (assumed to be proportional to restoration costs) for (a) the cumulative results and for (b) the best composite network, in both cases given increasing area targets (10; 100; 1,000; 10,000; 100,000 cells).

When restoration priorities were standardized by road density in order to account for the different restoration effort required in each cell, the location of priority areas changed but there was still a considerable overlap between the predictions from the cumulative and the best composite network (Fig 4).

In all cases, the most influential species were the brown bear (*Ursus arctos*), the red fox (*Vulpes vulpes*), the wild boar (*Sus scrofa*) and the grey wolf (*Canis lupus*) (S1 Table). In fact, the species most influencing on the aggregated results were those occupying large habitat areas and dispersing long distances (Table 2), which usually are generalist large species. However, the relative contribution of dispersal distance and habitat area changed with the aggregation technique (Table 2).

**Table 2:** Contribution of individual species dispersal distance and amount of habitat to the cumulative and composite network results. Contribution is expressed as standardized slopes of the linear regression. R^2^ = Variance explained by the regression model.

Aggregation type	Dispersal distance	Habitat amount	R^2^
**Cumulative**	0.085	0.072	0.716
**A**	0.049	0.100	0.391
**B**	0.017	0.017	0.667
**C**	0.020	0.017	0.705
**D**	0.021	0.017	0.709
**E**	0.414	0.416	0.616
**F**	0.166	0.096	0.798
**G**	0.192	0.101	0.807
**H**	0.199	0.104	0.808

Running the analysis on all species required a total of 402 hours and 20 minutes (~16.7 days) on a computer with two 2.53GHz processors and 48 GB of RAM. In contrast, the analysis on the composite network required 100 hours and 17 minutes (~4.2 days). The computational time of the PC metric increases very rapidly (almost exponentially) with the number of nodes. For this reason, the total time for the individual species analyses depends on the number of species being evaluated and their distribution range (related to the number of nodes to be considered for a given species). Therefore, the benefits of the composite network approach in terms of computational time are likely to increase considerably when larger assemblages of species are to be assessed over larger areas or at higher spatial resolutions than in our case study for Italy.

## Discussion

The application of connectivity principles in conservation planning has proven challenging because the concept of connectivity is intrinsically species-specific [41,42]. Each species perceives the environment in its own way, depending on the conditions that represent suitable habitat for the species, how species responds to landscape heterogeneity and matrix resistance, and the spatial scale at which the species perceives and uses the environment [41,43–45]. This scale can strongly differ from the human scale of perception and use. As a consequence, conservationists need to rescale the connectivity concept case-by-case, aware that no perfect solution exists to maximise the benefit for all species. Multispecies connectivity analyses are rare, and are generally performed either on each species separately (e.g. [30]) based on entire functional groups using more simple and generalizable metrics [28,29], or accounting for landscape resistance to movement but disregarding species-specific differences in terms of dispersal distances and population distribution [22–24]. To our knowledge, the one presented here is the first practical attempt at estimating connectivity at the scales of multiple species, and averaging across them to obtain a multi-scalar connectivity layer.

The European Union call for ecological restoration and implementation of green infrastructures is an opportunity that must be taken seriously and exploited with scientific rigour. Ecological restoration and defragmentation interventions need to be planned with scientific support, in order to maximise the benefit and make the best use of conservation funds. Rural areas are increasingly abandoned in Europe [46], and this trend is expected to increase in the coming decades providing opportunities for rewilding [47]. In Italy, farmland and pastures in the Apennines have been increasingly abandoned leading to natural vegetation restoration and increasing opportunities for wildlife. These trends provide opportunities for defragmenting the landscape by easing the implementation of green infrastructures.

The areas with higher restoration priority are concentrated along the Alps and the Appennines, also when the results are standardized by road density (i.e. required restoration effort). These areas have higher amount of natural habitat and are generally less disrupted by roads. The restoration priority of one cell is mainly due to the importance of that cell in the network, and the spatial interactions and functional synergies between the different cells need to be considered through a connectivity assessment such as the graph-based one we applied in this study. It is preferable to defragment areas that are not surrounded by a largely degraded matrix, so that the benefits of such restoration would go beyond the local area and would improve the connectivity of the whole network. In other words, increasing local permeability in areas with high road density but low probability of being reached by animals has a marginal effect on connectivity, whereas mitigating the barriers of roads in areas which are likely to be part of the main movement pathways of animals has a major effect on the connectivity of the entire habitat network.

Species do not equally contribute to the assemblage-level restoration priorities. Species with higher amount of habitat and dispersing long distances contribute more. One on hand, it is logical that species with wider ranges have more influence on the priorities for the entire network, because they are present in more cells and hence their preferences (suitability) are influencing in a larger proportion of the entire study area. On the other hand this result would tend to translate in a major influence of generalist species of least concern for conservation (e.g. red fox or wild boar in this study) compared to other narrowly-distributed or endemic species. To avoid this to happen, species can be weighted according to their conservation status during the aggregation procedure. In addition, the influence of each particular species on the restoration priorities may be assessed more locally, within the ranges in which those most endangered species occur, hence providing a more comparable outcome for particular species of interest. Long-dispersing species contribute more to the restoration priorities because they are able to move several cells (here 10 km) away from a given initial location, thereby being able to benefit from the permeability gains in many different areas, and not just those in which they are initially located.

In this study, we assumed all species to be equally negatively affected by roads, although species can actually manifest a variety of responses [48], and even show differential avoidance of roads depending on their behavioural state [49]. In some cases species can even benefit from roads because feeding on road kills, or because of the negative effect of roads on their predators’ populations [50]. However, a recent meta-analysis conducted by Rytwinski and Fahrig [51] showed that large-bodied mammals tend to be more susceptible to the negative effects of roads than other species.

All aggregation types provided high correlations with the cumulated species results, although some performed better than others. The aggregation procedure is dependent on the metric adopted. Here we aggregated a PC-based metric, whose intrinsic complexity prevents obtaining a perfect match with the cumulative results for species. This is because the PC metric jointly accounts for several aspects that are of importance for connectivity assessments: between-patch and within-patch connectivity, and, in the case of inter-patch connectivity, both direct and indirect dispersal pathways by accounting for the contribution of intermediate stepping stones that may facilitate movement between source and destination patches [27,36,52]. In particular, the indirect pathways mediated by stepping stones are largely dependent on local and global network configuration in each particular case and their behavior in an aggregated network cannot be predicted analytically from the indirect dispersal probabilities in the individual species networks. Because of this reason it is expectable that no single aggregated network can perform perfectly when compared to the more intensive connectivity analyses for many individual species. Despite these results, the performance of the best aggregation approach was very high given the analyzed correlations; its use can be hence recommended with confidence for a restoration application as the one we considered in Italy. Considering a simpler connectivity metric, or for instance considering only one of the three fractions in which the PC index can be partitioned [36,52], could allow to further increase the similarity between an aggregated network and the cumulative species results. This is however not generally advocated for several reasons. First, because we found that the results for the PC-based aggregation were satisfactory given the high correlations reported. Second, and more importantly, because such simplification may result in a partial, incomplete assessment of connectivity that for example disregards the acknowledged importance of stepping stones on species movements (e.g. [53–55]). Third, because such simplification may lead to problematic comparisons of different areas or through time, or may potentially provide misleading conservation or restoration priorities depending on the metric used [56]. It must be noted, however, that although a sum or an average over the results of individual species might be more commonly applied to obtain a consensus model for a species assemblage (e.g. [57,58]), there is no reason to believe that such procedure is the most correct one. The results based on a graph accounting for all species together, besides the reduced complexity and computational cost, might be equally reliable from an ecological and planning point of view.

### Conclusion

The biodiversity crisis and the limited funding available for conservation call for a shift from complex, time consuming and partial assessments for many or a few individual species to multispecies approaches. The framework proposed here allows to provide a good approximation for identifying important areas for defragmentation considering many species and reducing the computational demand, as well as the analytical and related planning complexity. Yet, the same or similar aggregation procedures could be applied for different scopes in connectivity analyses in order to reduce the computational effort and account for many species. The benefits of such an approach ultimately depends on the number of species considered and the number of nodes occupied by each single species. In fact, computational time increases almost exponentially with the number of nodes in the graph, and the analysis for only one species present in most or all network nodes would require the same time than running the composite network considering all species at once. Large scale analyses on large species assemblages, for instance, could benefit from implementing such a framework [12,29]. We foresee that such an approach can be used to generalize connectivity studies on large scales and large numbers of species for which a quantification of habitat and dispersal estimates are available, therefore significantly improving our ability to understand and manage complex and diverse networks of habitats from regional to global scales.

## Supporting Information

S1 Table Species list, dispersal distance estimates and species influence on network connectivity. List of species considered in this study, median dispersal distance estimated through statistical models, the reference for the predictive models used, and the mean proportion of *varPC* for the best composite (Aggregation F) and the cumulative results.
(DOCX)
