# Supplementary material for: A Composite Network Approach for Assessing Multi-Species Connectivity: An Application to Road Defragmentation Prioritisation
Source: PLoS One. 2016 Oct 21;11(10):e0164794. doi: 10.1371/journal.pone.0164794 (PMC5074507; doi:10.1371/journal.pone.0164794)
Supplement: S1 Table — List of species considered in this study, median dispersal distance estimated through statistical models, the reference for the predictive models used, and the mean proportion of varPC for the best composite (Aggregation F) and the cumulative results. (DOCX) [file pone.0164794.s001.docx]

**Table S1.** List of species considered in this study, median dispersal distance estimated through statistical models, the reference for the predictive models used, and the mean proportion of VarPC for the best composite (Aggregation F) and the cumulative results.

| **Species** | **Dispersal distance**  **(km)** | **Prop VarPC**  **(Composite)** | **Prop VarPC**  **(Cumulative)** | **Reference**  **(dispersal estimate)** |
| --- | --- | --- | --- | --- |
| *Canis aureus* | 11.44 | 0.10 | 0.09 | Whitmee & Orme 2012 |
| *Canis lupus* | 38.33 | 0.36 | 0.24 | Whitmee & Orme 2012 |
| *Capra ibex* | 11.05 | 0.00 | 0.00 | Santini et al. 2013 |
| *Capreolus capreolus* | 4.91 | 0.05 | 0.04 | Whitmee & Orme 2012 |
| *Cervus elaphus* | 29.09 | 0.11 | 0.08 | Whitmee & Orme 2012 |
| *Dama dama* | 4.40 | 0.03 | 0.02 | Whitmee & Orme 2012 |
| *Felis silvestris* | 8.00 | 0.04 | 0.03 | Whitmee & Orme 2012 |
| *Hystrix cristata* | 4.24 | 0.04 | 0.03 | Santini et al. 2013 |
| *Lepus corsicanus* | 3.95 | 0.03 | 0.03 | Santini et al. 2013 |
| *Lutra lutra* | 17.38 | 0.01 | 0.01 | Whitmee & Orme 2012 |
| *Lynx lynx* | 53.82 | 0.25 | 0.17 | Whitmee & Orme 2012 |
| *Martes foina* | 5.48 | 0.14 | 0.12 | Whitmee & Orme 2012 |
| *Martes martes* | 6.71 | 0.02 | 0.02 | Whitmee & Orme 2012 |
| *Meles meles* | 7.09 | 0.12 | 0.10 | Whitmee & Orme 2012 |
| *Mustela putorius* | 3.95 | 0.03 | 0.02 | Whitmee & Orme 2012 |
| *Rupicapra pyrenaica* | 6.88 | 0.00 | 0.00 | Santini et al. 2013 |
| *Rupicapra rupicapra* | 9.74 | 0.08 | 0.05 | Whitmee & Orme 2012 |
| *Sus scrofa* | 13.21 | 0.38 | 0.30 | Whitmee & Orme 2012 |
| *Ursus arctos* | 81.85 | 0.88 | 0.41 | Whitmee & Orme 2012 |
| *Vulpes vulpes* | 7.74 | 0.37 | 0.31 | Whitmee & Orme 2012 |
